# IsoVis – a webserver for visualization and annotation of alternative RNA isoforms

**DOI:** 10.1093/nar/gkae343

**Published:** 2024-05-06

**Authors:** Ching Yin Wan, Jack Davis, Manveer Chauhan, Josie Gleeson, Yair D J Prawer, Ricardo De Paoli-Iseppi, Christine A Wells, Jarny Choi, Michael B Clark

**Affiliations:** Department of Anatomy and Physiology, The University of Melbourne, Parkville, Victoria, 3010, Australia; Department of Anatomy and Physiology, The University of Melbourne, Parkville, Victoria, 3010, Australia; Department of Anatomy and Physiology, The University of Melbourne, Parkville, Victoria, 3010, Australia; Department of Anatomy and Physiology, The University of Melbourne, Parkville, Victoria, 3010, Australia; Department of Anatomy and Physiology, The University of Melbourne, Parkville, Victoria, 3010, Australia; Department of Anatomy and Physiology, The University of Melbourne, Parkville, Victoria, 3010, Australia; Department of Anatomy and Physiology, The University of Melbourne, Parkville, Victoria, 3010, Australia; Department of Anatomy and Physiology, The University of Melbourne, Parkville, Victoria, 3010, Australia; Department of Anatomy and Physiology, The University of Melbourne, Parkville, Victoria, 3010, Australia

## Abstract

Genes commonly express multiple RNA products (RNA isoforms), which differ in exonic content and can have different functions. Making sense of the plethora of known and novel RNA isoforms being identified by transcriptomic approaches requires a user-friendly way to visualize gene isoforms and how they differ in exonic content, expression levels and potential functions. Here we introduce IsoVis, a freely available webserver that accepts user-supplied transcriptomic data and visualizes the expressed isoforms in a clear, intuitive manner. IsoVis contains numerous features, including the ability to visualize all RNA isoforms of a gene and their expression levels; the annotation of known isoforms from external databases; mapping of protein domains and features to exons, allowing changes to protein sequence and function between isoforms to be established; and extensive species compatibility. Datasets visualised on IsoVis remain private to the user, allowing analysis of sensitive data. IsoVis visualisations can be downloaded to create publication-ready figures. The IsoVis webserver enables researchers to perform isoform analyses without requiring programming skills, is free to use, and available at https://isomix.org/isovis/.

## Graphical Abstract

**Graphical Abstract:**
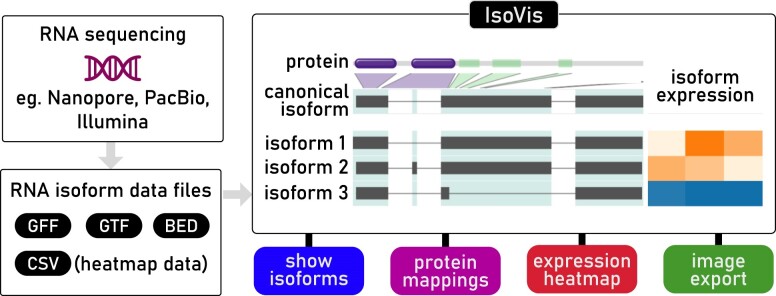

## Introduction

The transcriptome of higher organisms is immensely complex with genes commonly producing multiple mRNA products (transcript isoforms). For example, the current GENCODE 44 (1) catalogue of human genes annotates 252 835 transcript isoforms from 62 700 genes, with 89 067 of these isoforms expressed from 19 396 protein-coding genes. Studies in other species have found similar results (1–4). Alternate isoforms are generated by multiple processes including alternative transcriptional initiation sites, alternative splicing and alternative transcription termination sites, with >90% of human genes undergoing alternative splicing (5).

Alternative RNA isoforms encode different RNA and protein sequences, which can modify the functions of proteins and RNA. Specific isoforms play essential roles in numerous cellular, developmental and disease processes, including through genetic mutations that cause pathogenic changes to isoform expression (6,7). The recent maturation of long-read RNA-seq methodologies has also enabled more accurate isoform profiling from both bulk and single-cell samples (8). Studies using long-read RNA-seq have confirmed our understanding of isoforms remains far from complete and that many novel isoforms remain to be discovered in even the most well studied species (8–12).

The enormous number of known and novel isoforms in the transcriptome necessitates tools that can effectively visualise the isoforms identified from RNA-seq, allowing researchers to quickly understand which isoforms are expressed, how they differ from one another, and the potential consequences alternative isoforms could have on gene and protein function. Existing tools for RNA splicing or isoform visualization exist (13–23), however most are software packages that require expertise in R, Java, Python etc. to use (14–16,18,20,21). In addition, existing tools have limitations including: restriction to visualizing specific exons as opposed to whole isoforms (19,23); are not designed to visualize user data (22); can only display small numbers of isoforms or samples (14,16,18,19,23); don’t integrate isoform structure and expression levels (14,17,21); or don’t provide information about the impact of alternative isoforms on the encoded protein (13–16,21–23).

Here we introduce the Isoform Visualizer (IsoVis) to overcome these limitations. IsoVis is user-friendly and requires no programming skills to visualize data provided by the user. The intuitive design can display from 1-to-many isoforms in an isoform stack and their expression in a heatmap. IsoVis provides linked isoform and protein data, including the mapping of protein domains and motifs to exons, allowing changes to protein sequence and function between isoforms to quickly be established. The webserver can visualize isoform data from any species, with linked isoform, open reading frame (ORF) and protein domain information displayed for all chordate species in Ensembl plus additional popular model organisms.

## Materials and methods

### Implementation

IsoVis is a pure JavaScript application, built using Vue.js (https://vuejs.org/) and BootstrapVue (https://bootstrap-vue.org/). It has been implemented using the Nuxt 2 (https://nuxt.com/) framework for rapid development and deployment.

IsoVis reads the isoform data input file to parse the isoform information, including the locations of exons for each isoform. This is used to build the central visualization (‘isoform stack’), which shows one isoform per row. It then queries external databases to add additional information. Ensembl (24) is queried to fetch gene information as well as information on any known isoforms. InterPro (25) is queried to fetch protein domain and motif mapping information, which is visualized above the isoform stack. InterPro contains more protein data than can be visualized easily along a single row, hence key features of interest are extracted including Pfam-annotated domains, signal peptides (26), intrinsically disordered regions from MobiDB-lite (27) and coils (28).

Users can optionally upload a heatmap data input file. IsoVis parses the file and extracts data from the gene of interest selected by the user, which will then be used to build a heatmap showing the sample data of each shown isoform.

When users upload an isoform stack file containing data from more than one gene, IsoVis shows a prompt to select a gene for visualization. Users can search for the desired gene using either its gene ID specified in the isoform stack file or the Ensembl gene symbol associated with it. When users search via a gene symbol/ partial gene symbol, IsoVis queries the mygene.info service (https://mygene.info) (29) with this information and obtains a list of possible matches from the user-specified species. Then, it checks the Ensembl gene ID of each symbol in the list, and symbols that have matching IDs with those from the isoform stack file are displayed for the user to select from.

Since IsoVis is a pure JavaScript application, uploaded files are read and processed locally within the browser's memory. Hence no data is uploaded to, or stored on, the IsoVis server. As all data being viewed on IsoVis remains private to the user, IsoVis can be utilized for sensitive datasets that cannot be sent to, processed, or stored by, unauthorized third parties.

IsoVis is open source and users can create their own local install by downloading the source code from GitHub (https://github.com/ClarkLaboratory/IsoVis) and following the installation instructions provided.

## Results

Here we introduce IsoVis, an accessible and user-friendly webserver for the visualization of RNA isoform structure, expression and protein features. IsoVis accepts a variety of standard transcript and expression files as input, displays isoforms in a fast and intuitive design and outputs high-quality, publication-ready visualization images.

### Data upload and selecting species and genes to view

#### Required inputs

Stack data file: Users upload a GTF, GFF or BED file containing RNA isoform data into IsoVis. All BED file formats from BED4-to-BED12 are accepted. Heatmap data file: Users can optionally upload a CSV or tab-separated text file containing isoform expression levels in one or more samples. Input files must contain gene and transcript IDs and can contain RNA isoform data ranging from one gene to a whole transcriptome. Use of Ensembl gene and transcript IDs are recommended as these are required to fetch canonical isoform and protein data from Ensembl (24) and InterPro (25) and search via gene symbols. Detailed information on data input requirements is provided in the IsoVis website help section.

#### Species and gene selection

**Figure 1.**
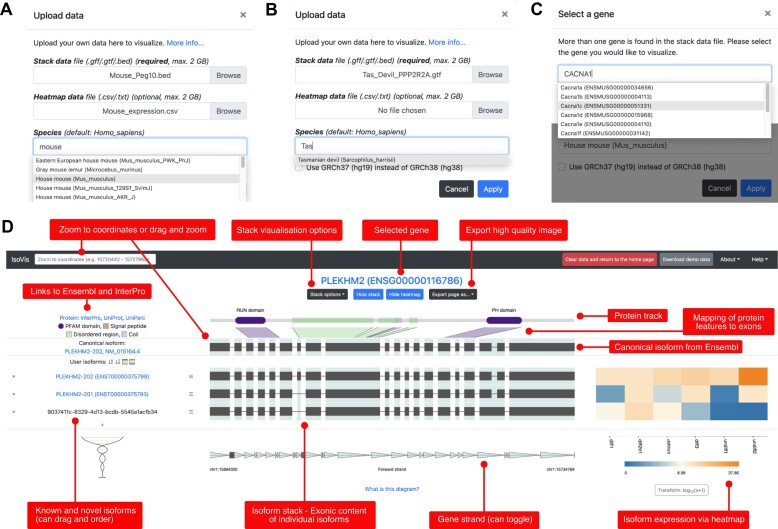
IsoVis Data upload and gene selection and visualization features. (**A**) Data upload function. Select stack (GTF, GFF or BED) file and optionally an expression heatmap (CSV or TXT) file. Search > 300 species to display linked information. (**B**) Species search includes all chordate species in Ensembl plus fruit fly and yeast. (**C**) Select gene of interest by Gene ID or gene symbol. (**D**) Screenshot of IsoVis gene isoform visualization page with annotation of features (red squares). Each row in central stack corresponds to an isoform, displaying splicing and exon structures, as well as expression values as a heatmap where this is available. Isoform/transcript IDs provided by the user are displayed on the left-hand side, with known IDs linked to their associated Ensembl pages. The canonical isoform is sourced from Ensembl and protein information for the gene is visually summarized and key features are mapped to exons on the canonical transcript. Many of the visual elements are interactive, including toggles to show open reading frames and to reverse the reading direction. Data from (30).

IsoVis can visualize properly formatted isoform stack and expression heatmap data from any species. IsoVis provides linked information from all chordate species (>300) in Ensembl (24), plus common model organisms *Drosophila melanogaster* (fruit fly) and *Saccharomyces cerevisiae* (Baker's yeast). Species is set to *Homo sapiens* by default. For all other supported species, selecting the correct species from the ‘Upload data’ species search box will generate the linked information (Figure 1A, B).

If a file contains 1 gene, it will be visualized by default. For files containing multiple genes, the user will be asked to select a gene of interest and may switch genes later. Gene selection can be performed by searching for gene IDs, and where there is a MyGene.info (29) gene symbol linked to the ID, by gene symbol (Figure 1C). The IsoVis visualization currently displays one gene at a time. Other genes from the stack can be selected by clicking on the ‘Change selected gene’ button on the visualization page.

### IsoVis features

#### Isoform Stack: visualization and comparison of isoform structures, splicing and exonic content

The central visualization in IsoVis is the Isoform stack (Figure 1D). Each row corresponds to an isoform. Exons are shown as black boxes and introns as thin lines. Green shading is used to identify any region that is exonic in any isoform, enabling quick assessment of where isoforms differ in structure and sequence. Introns are shrunk and normalized to focus on exonic content. Hovering the mouse/cursor over an exon within the stack displays additional information about the exon, including its exon number, exonic region and genomic coordinates. User provided transcript/isoform IDs are shown to the left of the stack.

The gene strand and 5′ and 3′ coordinates of the gene are displayed below the stack. The gene strand is labelled and indicated by arrowheads pointing from 5′ to 3′. Each arrowhead covers the same length of genomic sequence and therefore their compression gives a visual indication of how much each intron has been shrunk. Many aspects of the stack are interactive, or can be modified using the ‘Stack Options’ menu. Intron normalization can be toggled to show the stack in genomic scale. Reading direction can be reversed to show a gene from left-to-right or right-to-left as per user preference.

IsoVis has a zoom function to examine small scale changes between isoforms, such as changes to exon boundaries. Click and drag to select the region to zoom into or enter the precise genomic coordinates in the ‘Zoom to coordinates’ box. Zoom can be re-set by right clicking.

The order and number of isoforms shown is completely user customizable. By default isoform are displayed in the order found in the uploaded stack file. Isoforms can be dragged to re-ordered, or hidden from the stack using the ‘x’ button, while all isoforms can be hidden or shown by selecting the ‘+’ button to display the Edit Isoform list feature. Isoforms can also be sorted alphabetically or by mean expression level using the sort icons.

#### Heatmap: quantification and comparison of isoform expression levels within and between samples

IsoVis displays isoform expression data from each sample in a colour-blind friendly heatmap (Figure 1D). Hovering the cursor over a cell will display the isoform, sample and expression value. Log10 transformation of expression values can also be performed.

#### Canonical transcript and links to reference isoform data: comparison of experimental data to known gene isoform information

When uploaded data is labelled with gene and transcript IDs from a supported species Ensembl (24) and InterPro (25) are queried to display linked information (Figure 1D). The Ensembl canonical isoform for the gene of interest is imported and displayed above the isoform stack, allowing comparison of the annotated major isoform of the gene with those found in user samples. Gene and isoform labels are hyperlinked to their Ensembl pages. The open reading frame (ORF) within the canonical isoform and all annotated isoforms can be toggled on and off using the Stack Options menu, identifying which exons encode UTRs and CDS.

#### Mapping of protein domains and features to exons: visualizing the impact of alternative isoforms on proteins

Alternative isoforms can code for proteins with altered sequences and functions (6). To enable researchers to quickly estimate the possible impact of alternative isoforms on the encoded proteins IsoVis fetches protein domains and features from InterPro (25). The protein corresponding to the canonical isoform is displayed in a protein track at the top of the stack, with each feature mapped to the exonic regions which encode them. Summary descriptions of each protein domain are also provided (Figure 1D). This immediately allows the user to see, for example, if the gain or loss of an exon in an alternative isoform falls within a known protein domain and therefore might modulate or disrupt its function. Protein information is shown by default but can be disabled in the Stack Options menu.

#### High quality visualizations

The IsoVis Image Export feature allow users to download high-quality publication-ready images in PNG, JPEG, SVG or PDF formats.

### Use cases

#### Visualizing differentially expressed isoforms during cellular differentiation

**Figure 2.**
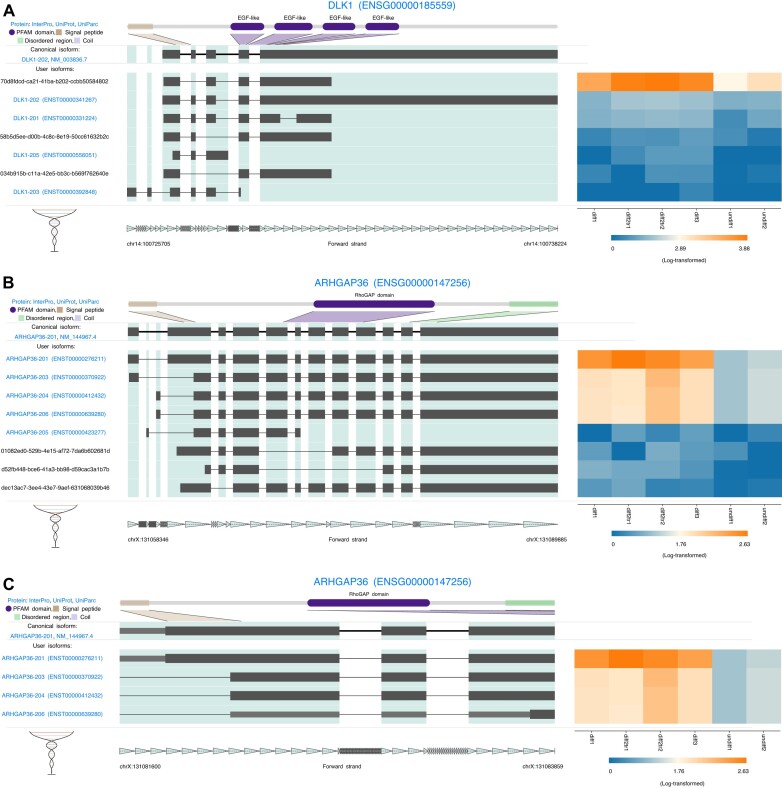
Differential expression of known and novel isoforms during SH-SY5Y differentiation (30). (**A**) Upregulation of a novel isoform of *DLK1* during differentiation. (**B**) Upregulation of 4 *ARHGAP36* isoforms during differentiation. (**C**) Zoom to exons 2–4 of differentially expressed isoforms from (B). An alternative exon boundary in isoforms 203, 204 and 206 causes them to lack the N-terminal signal peptide found in canonical isoform 201. ORF toggle causes exonic coding regions to be thicker and untranslated regions to be thinner. Protein domains (purple), signal peptides (brown), disordered regions (green).

Cellular differentiation leads to widespread gene and isoform expression changes. Previous investigation identified 326 differentially expressed isoforms during differentiation of SH-SY5Y neuroblastoma cells (30). *DLK1* is involved in regulating cell growth and differentiation (31). IsoVis reveals that while 7 *DLK1* isoforms were expressed, almost all expression was from a novel isoform with skipping of exon 2 that was upregulated during SH-SY5Y differentiation into neural-like cells (Figure 2A). *ARHGAP36* is reported to be a Rho GTPase activator and downstream effector of sonic hedgehog signalling (32). Four *ARHGAP36* isoforms were upregulated during SH-SY5Y differentiation (Figure 2B). Toggling known ORFs and zooming into the start of the coding region reveals 3 of the upregulated isoforms are missing the N-terminal signal peptide and so may have a different cellular localization to the canonical isoform (Figure 2C).

#### Isoform expression visualization in public large-scale datasets

**Figure 3.**
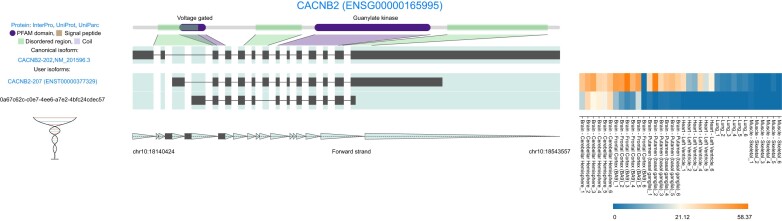
Novel cerebellum-specific *CACNB2* isoform in GTEx data (9). Protein domains (purple), disordered regions (green).

The Genotype-Tissue Expression (GTEx) consortium have profiled expression in hundreds of tissues and cell lines. We used IsoVis to visualize long-read RNA-seq isoform data from the GTEx V9 release, enabling investigation of the structure and expression of the >70 000 novel isoforms reported (9). *CACNB2* is a voltage-gated calcium channel (VGCC) subunit (33). GTEx data identified one known *CACNB2* isoform (*CACNB2-207*), which was expressed across different regions of the brain and heart and one novel isoform that was largely specific to the cerebellum and not expressed at all outside of the brain (Figure 3). This novel *CACNB2* isoform has an alternative transcriptional start site and skipping of canonical exon 7 but retains the VGCC beta and guanylate kinase domains.

#### Cell-type expression in single-cell datasets

**Figure 4.**
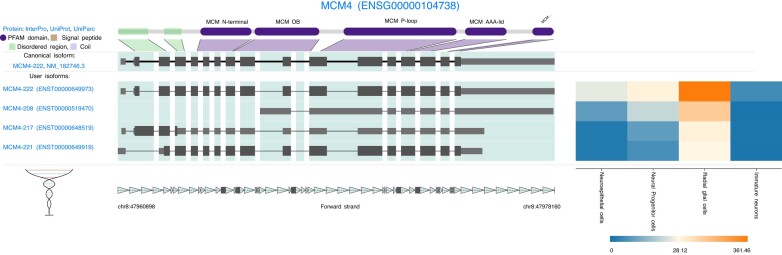
Single-cell isoform expression of *MCM4* during stem-cell to cortical neuron differentiation (34). For simplicity, expression from cells of the same cell type were pooled to create ‘pseudobulk’ expression values. Protein domains (purple), disordered regions (green).

Advances in single-cell RNA-seq (scRNA-seq) methods are enabling single-cell isoform profiling (8). Expression of isoforms across cells and cell-types can identify those involved in the functions of, or transitions between, cell types. We visualized differentially expressed isoforms from scRNA-seq of the differentiation of stem-cells to cortical neurons (34). Multiple DNA replication licensing factor *MCM4* isoforms were upregulated in radial glial cells, including the retained intron isoform *MCM4-208*, which is thought to be a non-coding transcript (24) (Figure 4).

### Demo datasets

IsoVis has an inbuilt demo data page showing the human gene *PLEKHM2* from nanopore direct RNA sequencing of SH-SY5Y cells (Figure 1D) (30). IsoVis also hosts whole transcriptome sequencing data from multiple studies, including all datasets shown as use cases (9,30,34), enabling users to fully explore the various features of IsoVis. All demo/hosted data are downloadable.

## Discussion and conclusion

Gaining a full understanding of the complexity of the transcriptome and making the most of RNA sequencing datasets requires going beyond gene counts to investigate RNA isoforms. The growing popularity of long-read RNA-seq approaches, which are specifically designed to output high-quality isoform-level data, further necessitates tools to analyse expression results at the isoform level. IsoVis is a novel tool for rapid visualization and analysis of RNA isoforms and is applicable to single-gene amplicon sequencing expression datasets through to whole transcriptome bulk or single-cell libraries. IsoVis has been designed to be applicable to a wide range of researchers by requiring no programming skills, being compatible with a wide range of species and keeping the data under analysis private to the user. In the future we plan to further increase the number of species IsoVis provides linked transcript and protein information for, while also expanding the quantity of protein features and ORF data provided.

IsoVis allows users to explore the rich isoform-level data produced by RNA-seq and provides an intuitive overview of how gene isoforms differ and diverge. Such information will accelerate the understanding of genes, cell types and tissues and facilitate hypothesis generation to investigate the potentially divergent functions of alternate gene isoforms.

## Data Availability

IsoVis is available at https://isomix.org/isovis/. This website is free and open to all users and there is no login requirement. IsoVis is a fully open source project, under the Mozilla Public License 2.0, and its source code can be found at https://github.com/ClarkLaboratory/IsoVis (doi: 10.5281/zenodo.10864822). The source code includes the demo data.

## Notes

Present address: Jarny Choi, St Vincent's Institute of Medical Research, Fitzroy, Victoria, 3065, Australia.
